# Edible Halophytes with Functional Properties: In Vitro Protein Digestibility and Bioaccessibility and Intestinal Absorption of Minerals and Trace Elements from Australian Indigenous Halophytes

**DOI:** 10.3390/molecules28104004

**Published:** 2023-05-10

**Authors:** Sukirtha Srivarathan, Rama Addepalli, Oladipupo Qudus Adiamo, Gethmini Kavindya Kodagoda, Anh Dao Thi Phan, Olivia Renee Louise Wright, Yasmina Sultanbawa, Simone Osborne, Michael Erich Netzel

**Affiliations:** 1 ARC Industrial Transformation Training Centre for Uniquely Australian Foods, Queensland Alliance for Agriculture and Food Innovation (QAAFI), The University of Queensland, Indooroopilly, QLD 4068, Australia; s.srivarathan@uq.edu.au (S.S.);; 2 Department of Biosystems Technology, Faculty of Technology, University of Jaffna, Ariviyal Nagar, Kilinochchi 44000, Sri Lanka; 3 Commonwealth Scientific and Industrial Research Organization (CSIRO), Agriculture and Food, St Lucia, QLD 4067, Australia; 4 School of Human Movement and Nutrition Sciences, The University of Queensland, St Lucia, QLD 4072, Australia

**Keywords:** halophytes, saltbush, samphire, protein, minerals, trace elements, in vitro digestion, bioaccessibility, intestinal absorption

## Abstract

Halophytes are considered emerging functional foods as they are high in protein, minerals, and trace elements, although studies investigating halophyte digestibility, bioaccessibility, and intestinal absorption are limited. Therefore, this study investigated the in vitro protein digestibility, bioaccessibility and intestinal absorption of minerals and trace elements in saltbush and samphire, two important Australian indigenous halophytes. The total amino acid contents of samphire and saltbush were 42.5 and 87.3 mg/g DW, and even though saltbush had a higher total protein content overall, the in vitro digestibility of samphire protein was higher than the saltbush protein. The in vitro bioaccessibility of Mg, Fe, and Zn was higher in freeze-dried halophyte powder compared to the halophyte test food, suggesting that the food matrix has a significant impact on mineral and trace element bioaccessibility. However, the samphire test food digesta had the highest intestinal Fe absorption rate, whereas the saltbush digesta exhibited the lowest (37.7 vs. 8.9 ng/mL ferritin). The present study provides crucial data about the digestive “fate” of halophyte protein, minerals, and trace elements and increases the understanding of these underutilized indigenous edible plants as future functional foods.

## 1. Introduction

Halophytes dominate saline coastal areas and inland arid and semi-arid soils, thriving in saline environments (i.e., 200 mM NaCl) where 99% of salt-sensitive plants (i.e., glycophytes) perish due to ion toxicity induced by high salinity [1,2]. In recent years, several halophytes have received significant scientific interest across the world as emerging novel foods, mainly as potential sources of protein, fiber, minerals, trace elements, and phytochemicals. The use of halophytes as vegetables, animal feed, and traditional medicines, as well as in environmental protection, phytoremediation, ornamental landscaping, hydrologic balancing, wildlife support, and industrial applications, represents alternative approaches for sustainable agriculture [2,3,4,5,6].

The micronutrients found in plants, such as minerals and other trace elements, help facilitate many functions in the human body. For instance, calcium (Ca) is involved in structural functions and blood clotting and plays a crucial role as an intracellular and extracellular regulator [7]. Similarly, potassium (K) is required for cellular functions, iron (Fe), zinc (Zn), and copper (Cu) act as enzyme cofactors and metalloproteins, and sodium (Na) and phosphorus (P) play roles as osmoregulators [8]. However, nutritional deficiencies in essential minerals affect almost one-third of the world’s population (i.e., 2 billion people) [9], despite minerals being essential for human health and being highly abundant in plants.

Bioaccessibility and intestinal absorption are important factors that determine the bioavailability and bioactivity of plant compounds by influencing the release and absorption of these compounds from digested food in the intestinal tract [10]. The bioaccessibility and intestinal absorption of ingested compounds, particularly minerals and trace elements, can vary widely depending on the nature of the compound and its interactions with the food matrix [11]. For instance, the bioaccessibility and intestinal absorption of minerals and other trace elements depend on the structure, nutrient interactions, and solubility of the compound, along with the absorption enhancers and inhibitors in the food matrix [12,13,14].

Bioaccessibility can be investigated in vivo or through in vitro methods [15,16] by mimicking human oral-gastrointestinal digestion. The common enzymes and salts used in in vitro digestion methods are pepsin, pancreatin, trypsin, chymotrypsin, α-amylase, and bile salts. These components are added to synthetic oral, gastric, and intestinal fluids and are combined with different foods or ingredients at 37 °C to facilitate oral-gastrointestinal digestion. In vivo, the released and partially digested nutrients are further metabolized by enzymes secreted by enterocytes in the intestine before being absorbed and circulated in the blood [13,17,18]. Therefore, in addition to in vitro oral-gastrointestinal digestion assays, cell culture models are also used to predict the intestinal absorption of digested nutrients from the digesta collected in vitro [17,19]. In particular, the Caco-2 human intestinal epithelial cell culture model has been commonly used for intestinal absorption studies as an alternative to animal models. Similarly, the human HT29-MTX-E12 cell line is also frequently used as a model of the gastrointestinal tract [11,20].

Research into halophytes and their potential applications as crops began around the 1950′s, although halophytes have been traditionally consumed across the world since ancient times [21]. For example, the *Atriplex* sp. (saltbush) and *Tecticornia* sp. (samphire) of halophytes have long been used for food and nonfood applications since ancient times in Australia [22,23]. Previous compositional analyses have revealed that saltbush and samphire are valuable sources of minerals (particularly K, Zn, Mg, Fe, and Ca) and protein [5,6]. However, studies investigating the bioaccessibility and intestinal absorption of samphire and saltbush nutrients, like protein and minerals, are very limited.

Therefore, the aim of the present study was to investigate the in vitro bioaccessibility and intestinal absorption of the protein and micronutrients in saltbush and samphire using simulated in vitro digestion and cell-based assays. The INFOGEST 2.0 static oral-gastrointestinal digestion method was used to prepare digesta from saltbush and samphire to determine bioaccessibility. Furthermore, standardized samphire and saltbush-based test foods were also prepared by mixing semolina paste and halophyte powder in a ratio of 1:1 to mimic a food matrix and its potential impact on bioaccessibility. The intestinal absorption of the target compounds was investigated using a Caco-2-HT29-MTX-E12 transwell membrane model following treatment with digesta. To date, this is the first comprehensive evaluation of the in vitro bioaccessibility and intestinal absorption of bioactive compounds, such as protein, minerals, and trace elements, from samphire and saltbush, as well as their respective test foods.

## 2. Results and Discussion

### 2.1. Halophytes as Sources of Protein and Amino Acids

#### 2.1.1. In Vitro Protein Digestibility of Halophyte Digesta Samples

Several studies have shown that halophytes contain significant levels of protein and essential amino acids [5,6,24,25] compared to other plant foods like rice and maize. However, protein type and nutritional quality determine digestibility, intestinal absorption, and the potential health benefits of dietary proteins and amino acids [26]. An in vitro protein digestibility assay was performed on the day the digesta samples were defrosted and filtered to avoid protein degradation through successive freeze-thaw cycles [27].

**Figure 1:**
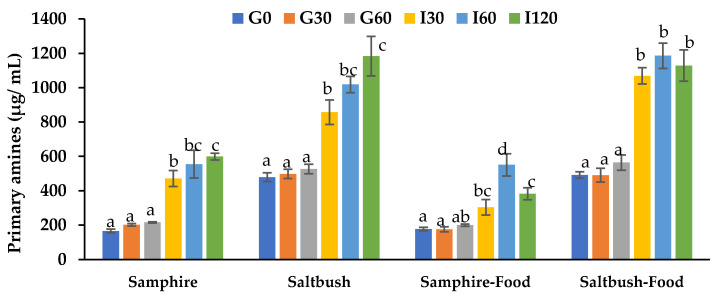
In vitro protein digestibility of samphire, saltbush, and test food digesta samples. Data are means ± SD (*n* = 6). G0, G30, and G60: gastric phase at 0, 30, and 60 min; I30, I60, and I120: intestinal phase at 30, 60, and 120 min. Means with different letters indicate significant (*p* < 0.05) differences.

In vitro protein digestibility was observed through the release of primary amines (µg/mL). As shown in Figure 1, samphire and the samphire test food had the lowest primary amine concentrations compared to the other samples. These findings were later confirmed by amino acid analyses (Table 1), where saltbush (87.3 mg/g DW) exhibited two times higher total amino acids compared to samphire (42.5 mg/g DW). Protein digestibility (PD) was also evaluated by calculating the percentage increase in the primary amines released across all sampling time points compared to G30 (Table 2). PD remained constant for samphire and saltbush throughout the gastric phase (G30–G60) before significantly increasing during the intestinal phase (I30). Similarly, the PD of test foods followed the same trend (Figure 1). However, the PD of samphire (197%) was greater than that of saltbush (138%), and the PD of samphire test food (118%) was less than saltbush test food (130%; Table 2).

Protein digestibility varies according to compositional differences in plant material, food processing methods, in vitro digestion methods, digestive enzymes, digestion duration, the method of PD employed, and the method used to calculate PD [28]. Additionally, peptidase was not included in the present study; therefore, dipeptides or oligopeptides may have persisted that would have otherwise been digested in vivo. Unlike other studies, total acid hydrolysis was not used in the present study to calculate PD; thus, comparisons cannot be made with PD studies that used acid hydrolysis. Although comparisons with other studies may be limited, the present study reveals the differences in PD among the freeze-dried halophyte powders and halophyte test foods.

Overall, samphire PD was higher than that of the saltbush samples despite having a lower protein content (7.6–12.6 g/100 g DW [5]) compared to saltbush (20.1 g/100 g DW [6]). Previous compositional analyses have revealed that samphire and saltbush are valuable sources of protein, fiber, and minerals and that the results are comparable with baby spinach, a popular vegetable in many countries [5,6]. Samphire samples may contain proteins with different structures and fewer anti-nutrients, such as tannins, phytates, and trypsin inhibitors, which may decrease PD. These anti-nutrients have been reported to bind to and reduce the bioaccessibility of other nutrients, such as minerals and proteins [29,30]. Moreover, the sample matrix and protein solubility also influence in vitro PD [31].

#### 2.1.2. Amino Acid Profile of Halophyte Samples

The nutritional value of dietary proteins depends on amino acid composition. Therefore, acid hydrolysis and UHPLC detection of free amino acids (AAs) were used to assess the AA composition of samphire and saltbush. Acid hydrolysis facilitates AA release from proteins and peptides by cleaving the amide bond; however, the final estimation of AAs is influenced by differences in peptide bond cleavage efficiency and by the acid stability of certain amino acids. For example, some amino acids (cysteine and tryptophan) are unstable during acid hydrolysis, while other amino acids (valine, isoleucine, and leucine) contain peptide bonds that are resistant to hydrolysis [32,33]. Thus, the method used to measure AA in this study may not have detected all AAs present.

The total AA contents in samphire and saltbush were 42.5 mg/g DW and 87.3 mg/g DW, respectively, higher than levels recently reported for other edible halophytes like *Suaeda aegyptiaca* (1.2 mg/g DW) and *Suaeda monoica* (1.2 mg/g DW; [34]). The main AAs found in the samphire and saltbush species were glutamic and aspartic acids. The high abundance of these AAs might be attributed to the important role these AAs play in adapting to salinity stress. Halophytes respond to saline stress through the production of stress proteins and the accumulation of some free AAs (like glutamine, asparagine, proline, tyrosine, alanine, arginine, and glycine), non-structural carbohydrates, and betaines [35,36]. The abundance of acidic and neutral AAs, like glutamic acid, aspartic acid, leucine, and glycine, in samphire and saltbush species was also observed in another halophyte *Salicornia herbacea* [37].

The total essential amino acids (EAA) in samphire and saltbush varied between 40–43% of the total AAs and was higher than the EAA abundance reported in other halophytes (*Mesembryanthemum crystallinum*, *Crithmum maritimum*, and *Triglochin maritima*: 36 vs. 37 vs. 27%) [35]. However, the EAA content of saltbush (37.4 mg/g DW) was similar to another halophyte, sea fennel (*C. maritimum*: 41.7 mg/g DW), as reported by Sánchez-Faure and others [35]. Among the EAAs, leucine (21.6%) and valine (16.3%) were the predominant EAAs in the samphire species, whereas leucine (20.8%) and lysine (16.9%) were the most abundant EAAs in saltbush. When compared to the total AAs, the content of lysine in saltbush was higher (6.3 mg/g DW and 7% of total AAs) than whey protein (2.6%; [38]). The high content of leucine, isoleucine, and valine in plant foods is important as these branched chain AAs are involved in protein synthesis turnover and signaling and glucose metabolism in humans [39].

Like legumes, halophytes contain low amounts of sulfur-containing AAs. For example, saltbush had 0.8 mg/g DW methionine, while the methionine content was below detectable levels (LOD; 0.6 mg/g) in samphire. The high lysine content and low sulfur AA content could make these halophytes ideal ingredients for cereal products, as cereals often have high sulfur AA and low lysine contents; therefore, halophytes might help balance the supply of EAAs in the human diet.

### 2.2. Halophytes as Sources of Minerals and Trace Elements

The consumption of micronutrients, such as minerals and trace elements, is essential for maintaining human health. The total concentration of minerals and trace elements investigated in the present study was used to calculate bioaccessibility.

#### 2.2.1. In Vitro Bioaccessibility of Minerals and Trace Elements

**Figure 2:**
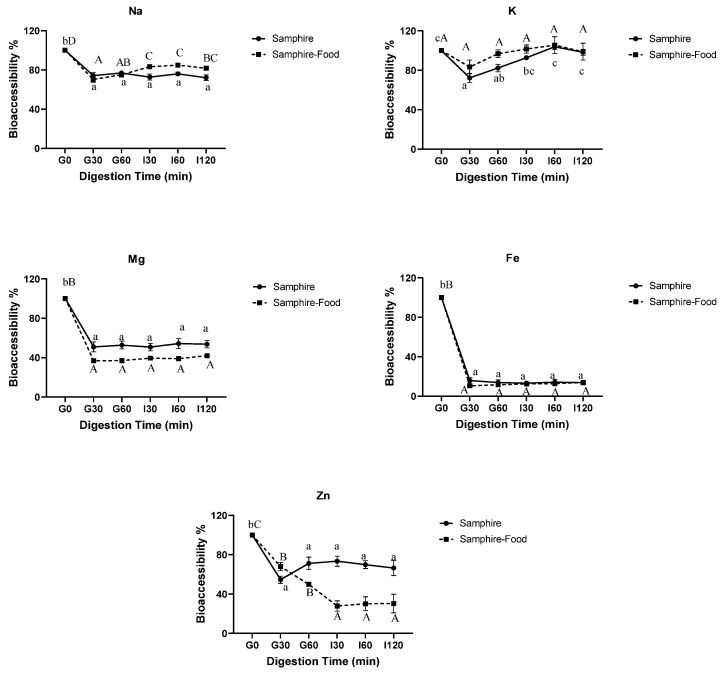
In vitro bioaccessibility of selected minerals and trace elements in samphire and samphire test food digesta samples. Data are means ± SEM (*n* = 6). Means with different letters indicate significant (*p* < 0.05) differences.

**Figure 3:**
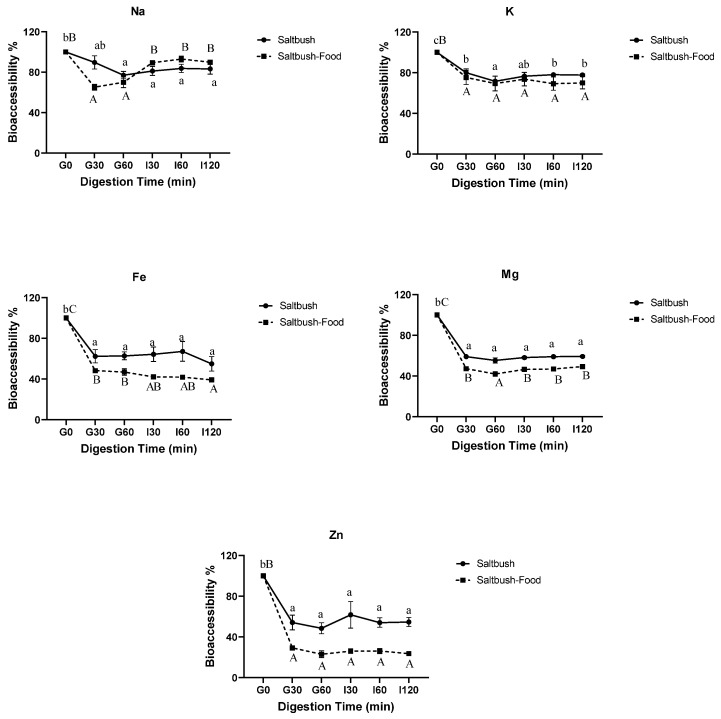
In vitro bioaccessibility of selected minerals and trace elements in saltbush and saltbush test food digesta samples. Data are means ± SEM (*n* = 6). Means with different letters indicate significant (*p* < 0.05) differences.

In Figure 2, the bioaccessibility of the divalent cations (Mg, Zn, and Fe) in samphire and the samphire test food digesta significantly (*p* < 0.05) decreased from G0 to G30 and remained significantly lower throughout gastrointestinal digestion. The bioaccessibility of the monovalent cations (Na and K) was different from the trends observed for the divalent cations. The potassium levels did not change in the samphire-only digesta; however, the K levels in the samphire test food digesta significantly decreased throughout the gastric phase before increasing in the intestinal phase back up to G0 levels. The sodium levels in all samphire digesta significantly decreased from G0 to G30 and remained relatively constant from G30–I120. In Figure 3, the bioaccessibility of the divalent (Mg, Zn, and Fe) and monovalent (K) cations in saltbush and the saltbush test food digesta significantly (*p* < 0.05) decreased from G0 to G30 and remained significantly lower throughout gastrointestinal digestion (i.e., G60-I120).

Overall, the halophyte freeze-dried powders showed higher divalent cation bioaccessibility (Mg, Fe, and Zn) than the halophyte test foods, suggesting that the food matrix impacted mineral bioaccessibility [12,14]. Conversely, the Na and K bioaccessibility was similar between the halophyte test foods and halophyte freeze-dried powders (Figure 2 and Figure 3), although bioaccessibility widely varied between the samples from G0 to I120. Additionally, the mineral present in the highest amount in the samphire gastric digesta was K, followed by Na, Zn, Mg, and Fe. The same trend was observed in the intestinal phase. These observations could be attributed to the presence of anti-nutrients (e.g., phytates, oxalates etc.). In addition to phytates, previous studies have revealed that saltbush and samphire also contain oxalates, trypsin inhibitors, saponins, and hydrolyzable tannins. However, the results clearly showed that the content of these anti-nutrients was lower than that in baby spinach, except for hydrolyzable tannins [5,6]. According to Marolt and others, the formation of metal-phytate complexes is more likely with divalent cations than with monovalent cations [40]. In addition to total ion charge, the formation of metal-phytate complexes depends on other factors, such as molar ratio, pH conditions, and the protonation of phytate ligands [40]. These findings are similar to the bioaccessibility of *Sarcocornia ambigua*, a plant belonging to the family Amaranthaceae, reported by Bertin, Maltez, Gois, Borges, Borges, Gonzaga, and Fett [13], who found that the most bioaccessible mineral in *S. ambigua* was K, followed by Mg and Zn. The most bioaccessible mineral in the saltbush gastrointestinal digesta was Na, followed by K, Fe, Mg, and Zn.

Iron deficiency anemia affects approximately one-third of the world’s population and is common among adolescent girls, infants, and pregnant women in developing countries [41]. Increased demand for Fe during growth and reproduction is intensified by poor dietary intake and the bioavailability of Fe from foods [42]. Similarly, Zn deficiency is also prevalent and estimated to affect more than 3 billion of the world’s population [43]. Both Fe and Zn deficiencies coexist in populations that consume plant-based foods as the main source of these minerals and other trace elements. Consequently, the fortification and supplementation of Fe and Zn are practiced in many countries to prevent such deficiencies [44,45]. However, iron and zinc compete for similar transport pathways, limiting the intestinal absorption of both trace elements [46]. This should also be considered when developing efficient supplementation and fortification strategies.

Several underutilized plants have gained significant attention as emerging food sources of essential minerals and trace elements [6]. Samphire and saltbush are two such plants and are known to be valuable sources of minerals and trace elements [5,6]. In this study, the Fe content of samphire (1565.5 mg/kg DW) was found to be superior to saltbush (41 mg/kg DW), although in vitro Fe bioaccessibility was lower in samphire compared to saltbush (i.e., 13.7% vs. 62.1% bioaccessibility), indicating that Fe bioaccessibility may not be dependent on concentration. The bioaccessibility of minerals and trace elements may be reduced or increased according to the interactions with other molecules released during digestion. This could be explained by the presence of phytate, oxalate, and polyphenols, which form insoluble compounds and impair Fe bioaccessibility [14]. Other studies have reported similar trends in the bioaccessibility of Fe in beans, lentils, and cereals [47,48,49]. Similarly, saltbush has a higher Zn content (2.81 mg/kg DW) than samphire, although the bioaccessibility of Zn in saltbush was lower than samphire (i.e., 56.8% vs. 70.0% bioaccessibility). When the Ca and Fe bioaccessibility values of samphire and saltbush were compared with respect to overall Ca content, interesting trends were observed. As explained by Anderson, calcium in food may positively impact Fe bioaccessibility. This may be due to the formation of Ca-phytate complexes that prevent Fe from binding phytate, allowing it to be more bioaccessible [50]. Hence, a higher Ca content in saltbush (1.7 vs. 1.2 g/100 g DW) may explain the higher Fe and lower Zn bioaccessibility in saltbush compared to samphire.

Furthermore, the higher Zn bioaccessibility observed in the samphire samples may be attributed to the formation of more soluble Zn complexes. For instance, digested samphire releases peptides or amino acids that can form soluble complexes with Zn, subsequently increasing Zn bioaccessibility due to improved cation solubility [51]. According to Joshi, Thatte, Prakash, and Jyothi Lakshmi [45] and Maares and Haase [51], histidine, cysteine, and glycine can positively impact the bioaccessibility of both Fe and Zn. This might be explained by the findings in the present study, where samphire PD was higher than saltbush PD (Table 2); subsequently, samphire Zn bioaccessibility was higher than saltbush.

Total mineral content does not always reflect intestinal mineral absorption; therefore, mineral bioaccessibility should be considered when determining the contribution of halophyte freeze-dried powders to the recommended dietary allowance (RDA) of minerals. In a previous sensory study (unpublished), 1 g of samphire freeze-dried powder that was tested as a salt substitute delivered 4.8% of the suggested sodium (96 mg) dietary target (SDT; [52]: 2000 mg/day;), 2.7% (0.2 mg) of the RDA (8 mg/day; [53]) for iron, 1.2% (4.1 mg) of the estimated average requirement (EAR; 0.35 g/day; [53]) for magnesium, 0.2% (0.01 g) of the adequate intake (AI; 4.7 g/day; [53]) of potassium, and 0.1% (0.01 mg) of the RDA (11 mg/day; [53]) for zinc. These values are similar to those reported by Bertin, Maltez, Gois, Borges, Borges, Gonzaga, and Fett [13] for *S. ambigua,* another herbal salt. Similarly, in a previous sensory study involving saltbush as a salt substitute, 1.4 g of saltbush freeze-dried powder delivered 5.1% (101 mg) of the suggested dietary target (SDT) for sodium, 0.44% of the RDA for iron (0.04 mg), 2.1% of the EAR for magnesium (7.6 mg), 0.9% of the AI for potassium, and 0.2% of the RDA for zinc (0.02 mg). However, mineral bioaccessibility is influenced by food matrices, food processing conditions, and intestinal pH.

When compared to saltbush (41 mg/kg DW), samphire is considered a good source of non-heme iron (1565.5 mg/kg DW), delivering almost 100% of the RDA for iron for adult men (i.e., 8 mg) in a 20 g “fresh-serving” (7.8 mg) (unpublished). The iron content of samphire in the current study is higher than in previously published data for the same species (101.4–414.6 mg/kg DW [5]), and this is most likely due to differences in the growing location, harvest procedure, and storage and transport conditions [6]. Even though samphire is considered a good source of iron, the absorption of non-heme iron (1–10; [54]) is less efficient than heme iron (15–35%; [55]) from animal tissue, presenting challenges for maintaining iron levels, as more people move towards plant-based diets. Therefore, it is important to understand intestinal iron absorption from different plant sources and identify ways to enhance non-heme iron uptake.

#### 2.2.2. In Vitro Intestinal Absorption of Minerals and Trace Elements

In vitro oral-gastrointestinal digestion was combined with the Caco-2-HT29-MTX-E12 transwell model to determine in vitro intestinal absorption as an indicator of trace element bioavailability from halophyte and halophyte test foods. Cell viability was measured with the CyQUANT Cell Proliferation Assay to ensure the treatment concentrations of halophyte and halophyte test food digesta samples were non-cytotoxic to Caco-2 and HT29-MTX-E12 co-cultures.

##### Effects of Halophyte Digesta Samples on Cell Viability

**Figure 4:**
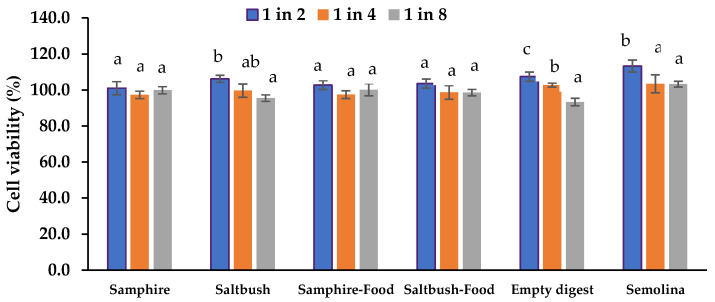
Effects of the halophyte digesta samples on Caco-2 and HT29-MTX-E12 co-culture cell viability in vitro using CyQUANTR cell proliferation assay. Data are means ± SEM (*n* = 6). Cell viability is expressed as a percentage compared to the HBSS control. Empty digests: digestive electrolytes and enzymes; Semolina: semolina, digestive electrolytes, and enzymes. Means with different letters indicate significant (*p* < 0.05) differences.

Cell viability was not significantly impacted by any of the filtered and diluted 60′ intestinal digesta samples (I60) (Figure 4). Hence, the lowest dilution (2:1) was selected for in vitro intestinal absorption assays.

##### Intestinal Absorption of Halophyte Minerals and Trace Elements

Intestinal absorption, expressed as the apparent permeabilities of halophyte minerals and trace elements, is shown in Table 3. Studies suggest that *P*app values of >1 × 10^−6^ cm/s and <1 × 10^−7^ cm/s (in the apical chamber (AC) to basolateral chamber (BC) direction) represent high and low permeation, respectively [56,57]. In this study, almost all the halophyte and halophyte digesta samples displayed high permeation for the tested minerals and trace elements. From the *P*app observed in this study, the monovalent cations (Na and K) exhibited higher intestinal absorption compared to the divalent cations (Mg, Zn, and Fe). This observation could be attributed to the presence of anti-nutrients (phytates, oxalates, etc.) and the fact that monovalent cations are more hydrophilic than divalent cations. Similarly, the intestinal absorption of Na, K, and Mg from the halophyte food digesta showed similar or higher rates to that of the halophytes alone, suggesting the positive impact of the semolina food matrix on intestinal absorption. The intestinal absorption of Fe and Zn from the halophyte test foods was lower than the halophytes alone, although the differences were not deemed significant (Table 3).

Overall, the *P*app of K, Mg, Zn, and Fe significantly varied across the different samples, except for Na, where *P*app did not significantly vary across the tested samples. However, the *P*app of Na from both the samphire and samphire test food digesta samples was higher than saltbush. Similarly, the samphire and samphire digesta samples showed higher intestinal absorption rates for Mg and Zn. The highest intestinal absorption of Zn in the samphire digesta samples was possibly due to the enhancing effect of a greater PD, as explained by Lombardi-Boccia et al. [58], who reported the enhancing effect of globulin protein on Zn bioavailability in beans. However, the absorption of Fe did not significantly vary across the digesta samples. Ferritin formation in vitro is considered a reliable indicator of iron absorption by Caco-2 cells and offers a useful prediction of Fe bioavailability from different foods when compared to other methods that rely on radioisotopes. Hence, ferritin production was also measured in response to halophytes and halophyte test foods and is included in the next section.

##### In Vitro Intestinal Iron Absorption Measured through Cellular Ferritin Production

In vitro ferritin production via Caco-2 cells or other cells representing absorptive human enterocytes is often used as a measure of intestinal iron uptake [59]. Similarly, the intestinal uptake of iron from the digesta samples was measured through Caco-2/HT29-MTX-E12 cellular ferritin production.

**Figure 5:**
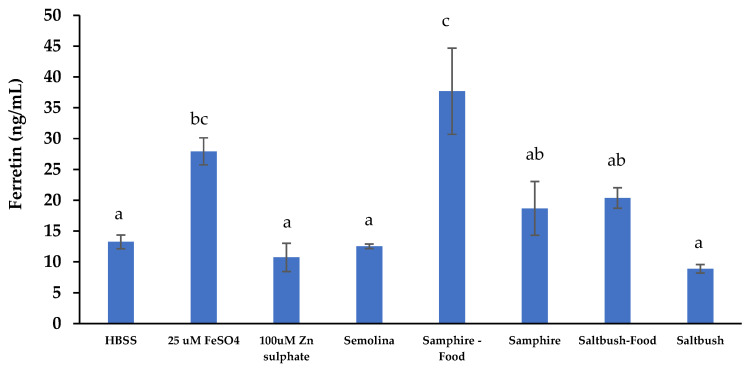
Iron uptake by Caco-2 and HT29-MTX-E12 co-culture cells from samphire, saltbush, and test food digesta samples. Data are means ± SEM (*n* = 3). Means with different letters indicate significant (*p* < 0.05) differences. Semolina: semolina, digestive electrolytes, and enzymes only; HBSS: Hank’s balanced salt solution.

In Figure 5, ferritin production in response to the samphire test food was the highest (37.7 ng/mL) compared to saltbush digesta, which produced the lowest level of ferritin (8.9 ng/mL). Adding samphire to semolina significantly increased ferritin production when compared to semolina alone. Similarly, adding saltbush to semolina also increased ferritin production; however, the increase was not significant. Ferritin production in response to 25 μM FeSO_4_ and HBSS served as the positive and negative controls and was significantly different. In contrast to the bioaccessibility results, the samphire digesta samples showed comparatively higher intestinal iron uptake (*p* < 0.05) than the saltbush digesta samples (Figure 5). Similarly, intestinal iron uptake from the samphire test food digesta was significantly higher than that of the saltbush test food digesta. The filtered extracts from saltbush digesta/saltbush food digesta had higher iron bioaccessibility, although these trends were not observed in ferritin production. This may be due to the presence of iron chelates or ligands limiting intestinal iron absorption [41,60]. Statistical comparisons showed that all halophyte digesta samples stimulated intestinal iron uptake more than or similar to the positive 25 μM FeSO_4_ control, except for saltbush.

Many different compounds in foods can inhibit or enhance intestinal non-heme absorption. In general, fiber, polyphenols, and anti-nutritional factors, including phytic acid, negatively impact the solubility, bioaccessibility, and intestinal absorption of non-heme iron [61]. Conversely, ascorbic acid weakly chelates and reduces non-heme iron, enhancing intestinal iron absorption [62]. Humans cannot synthesize ascorbic acid, so it must be consumed in the diet [63]. The consumption of fruits and vegetables creates a constant level of ascorbic acid in the intestinal lumen that promotes the absorption of non-heme iron; therefore, ascorbic acid was added to the samples in the present study, including the positive and negative controls, to mimic the levels of ascorbic acid normally present in vivo and facilitate intestinal iron uptake by the Caco-2 and HT29-MTX-E12 cells. Previously, Khoja and others reported increased intestinal iron absorption from fenugreek (sprouts and seeds), baobab (pulp), and moringa (leaves) when ascorbic acid was applied to digested extracts prior to Caco-2 cell treatment [41].

Samphire (6.2 mg phytic acid (PA)/g DW) and saltbush (6.2 mg PA/g DW) species have the same levels of phytic acid. Further, saltbush (65.6 mg/100 g DW) and samphire (10.6 mg/100 g DW) contains Vitamin C, but at different levels, as determined by [6] using a Waters UPLC-PDA system. Although the iron in saltbush was found to be more bioaccessible and soluble, the intestinal iron absorption from samphire was greater than saltbush, and this may have been due to lower molar ratios of phytic acid:iron [64]. For example, the phytic acid:iron molar ratio in samphire is 0.35:1 (e.g., 9.4 mmol PA: 26.8 mmol Fe), which is 38-fold lower than the phytic acid:iron molar ratio in saltbush (13.4:1). Low phytic acid:iron molar ratios, particularly ratios below 0.4:1, confer better intestinal iron absorption. Even though the molar ratio of vitamin C:iron in saltbush is higher than samphire (5.3 vs. 0.02), the higher phytic acid:iron molar ratio may prevent vitamin C from stimulating intestinal Fe absorption from saltbush [64,65]. As reported by Miller and Berner [60], the presence of soluble ligands or chelators, the molecular weight of iron, and the food matrix are the determinants of iron bioavailability from different foods. Therefore, further research into the impact of non-heme iron absorption enhancers and inhibitors is essential to maximize the potential of halophytes and other underutilized plants or plant products.

Increased cell ferritin is evidence that Fe has entered the cells since ferritin is produced in response to increases in intracellular iron [59]. Although the results show that the amount of bioaccessible Fe was highest in saltbush and the saltbush test food digesta, ferritin production in response to saltbush was lower than in the samphire digesta samples. A similar observation was found by Glahn et al. [66], who used the Caco-2 transwell model to show that the basolateral chamber (BC) became depleted of Fe following increasing Fe treatment of the cells in the apical chamber. These results suggested that the cells in the apical chamber (AC) reabsorbed the Fe that was transported into the BC [66]. A similar finding was also observed and explained by Beard et al. [67]. Since Fe homeostasis is a two-way process, the low Fe concentration in the BC of the samphire treatments (Table 3) in the study may be due to the rate at which Fe was transported from the AC to the BC and reabsorbed from the BC to the AC. Conversely, other studies report that ferritin formation by Caco-2 cells is proportional to the concentration of absorbable Fe in the culture medium [68,69].

## 3. Materials and Methods

### 3.1. Materials

A total of 1 kg samphire (*Tecticornia* sp.) was acquired from the Indigenous Community of Twin Lakes Cultural Park (Kimberley, WA, Australia), and 1 kg saltbush (*Atriplex* sp.) was sourced from Flinders Island (Victoria, Australia). Freeze-dried ground samples were stored at −35 °C. All analytical grade chemicals and standards were obtained from Sigma-Aldrich (Castle Hill, NSW, Australia). Semolina (sostanza) was purchased at a local supermarket in Brisbane, QLD, Australia.

### 3.2. Sample Preparation of Samphire and Saltbush Test Foods

Semolina was selected as a representative food matrix to prepare halophyte test food samples as it provides a neutral flavor and consistent texture. Semolina paste was prepared by adding boiling water to semolina at a ratio of 4:1, as described by Smyth [70], before being cooled at room temperature (RT). Both samphire and saltbush test foods were prepared with a 1:1 (semolina: halophyte) ratio.

### 3.3. In Vitro Simulated Oral and Gastrointestinal Digestion

Simulated oral and gastrointestinal digestion was performed on the samphire and saltbush samples using the static INFOGEST 2.0 method as described in Brodkorb et al. [71] with modifications. The enzymatic activities of amylase and pancreatin stocks were measured according to the protocols described in Ménard et al. [72]. The addition of enzymes was determined based on enzyme activity. Briefly, 0.5 g samples (halophytes freeze-dried powder or halophyte test food) were mixed with 1.25× simulated salivary fluid (SSF; 4 mL) at pH 7, 0.3 M CaCl_2_ (25 μL), 250 μL of salivary α-amylase (1497.4 U/mL) and Milli-Q water (725 μL) and incubated at 37 °C for 2 min in oral phase. For the gastric phase, oral digesta (5 mL) pH was adjusted to 3.0, using 6 M HCl, and mixed with simulated gastric fluid (SGF; 1.3 mL), 0.3 M CaCl_2_ (2.5 μL), 555 μL (3605.9 U/mg) of pepsin and Milli-Q water (3.14 mL), and incubated at 37 °C for 30 min. Gastric digesta samples were collected at 30 min (G30) with the remaining digesta incubated at 37 °C for another 30 min before another gastric digesta sample was collected at 60 min (G60). For the intestinal phase, the remaining 60 min gastric chyme (8 mL) was adjusted to pH 7 with 5 M NaOH, mixed with 7.5× simulated intestinal fluid (SIF; 65 μL) and 317 μL of 240 mM bile salts (activity: 505 mM), and incubated at 37 °C for 30 min. Next, 0.3 M CaCl_2_ (10 μL), 7.2 mL porcine pancreatin (11.1 U/mg), and Milli-Q water (402 μL) were added with the resulting mixture incubated at 37 °C for 120 min. Digested samples were collected at t = 30 (I30), t = 60 (I60) and t = 120 (I120) min in the intestinal phase. Enzymes were inactivated by snap freezing on dry ice, and the digesta samples were stored at −80°C. Empty digestion (i.e., without sample) was also included as a control.

### 3.4. In Vitro Protein Digestibility

In vitro protein digestibility was observed as the release of primary amines using the OPA (o-phthaldialdehyde) method [73]. Briefly, halophyte digesta samples were centrifuged at 10,000× *g* for 5 min at 23 °C with supernatants decanted and used for determining protein digestibility (PD). Absorbance was measured at 340 nm with methionine as the standard (250 μg/mL). PD was calculated using equation 1, and results were expressed as μg primary amines per mL of digesta supernatant (μg/mL).

Protein digestibility (PD) = Cs − Ce(1)

Cs and Ce are the final concentrations of primary amines in the sample digesta and empty digesta, respectively.

### 3.5. Analysis of Amino Acids

Amino acid composition of freeze-dried halophyte samples (0.1 g) was determined using hydrolysis with 6 M HCl at 110 °C for 24 h. After hydrolysis, all amino acids were analyzed using the Waters AccQTag Ultra chemistry methodology on a Waters Acquity UPLC system at the Australian Proteome Analysis Facility, National Collaborative Research Infrastructure Strategy (NCRIS), Macquarie University, New South Wales, Australia. Samples were analyzed in duplicate, and results were expressed as mg/g DW of the sample.

### 3.6. Cell Culture

Human Caco-2 and HT29-MTX-E12 cells were obtained from the American Type Culture Collection (ATCC) (Manassas, VA, USA) and Sigma-Aldrich (Castle Hill, NSW, Australia), respectively. Both Caco-2 and HT29-MTX-E12 cell lines were grown in Dulbecco’s Modified Eagle Medium (DMEM) supplemented with fetal bovine serum (FBS; 10% (*v*/*v*)), non-essential amino acids (NEAA; 1×), penicillin (100 U/mL), streptomycin (100 μg/mL), and glutamax (2 mM) in vented culture flasks at 37 °C and 5% CO_2_. Cells were grown to 90% cell confluency before passaging, and cell assays were performed using cells at passages 10–20.

### 3.7. Cell Viability Studies

To ensure all sample concentrations applied to cells were non-cytotoxic, cell viability assays were conducted using the CyQUANT Cell Proliferation Assay. A 9:1 mixture of Caco-2 cells and HT29-MTX-E12 were grown in 96-well black plates at a cell density of 9 × 10^4^ Caco-2 cells/mL and 1 × 10^4^ HT29-MTX-E12 cells/mL, as previously described by Akter, Addepalli, Netzel, Tinggi, Fletcher, Sultanbawa and Osborne [11]. Co-cultures were grown for 1 week at 37 °C with 5% CO_2_, with growth media changed every 2–3 days. After 7 days, culture media was discarded, and replaced with Hank’s balanced salt solution (HBSS; 100 μL), followed by incubation for 2 h. Prior to cell treatment, digesta samples were centrifuged at 10,000× *g* for 5 min before being filtered through 10 kDa molecular weight cut-off filters (14,000× *g* for 10 min). Next, HBSS was removed and replaced with 100 μL filtered and diluted (1/2, 1/4, and 1/8 using HBSS). HBSS alone was added to the control wells. Plates were incubated overnight (16–18 h) at 37 °C and 5% CO_2_ before digesta samples were removed, and the cells washed with sterile 100 μL HBSS. Next, 100 μL 1× CyQUANT^R^ NF dye solution diluted in HBSS (1:500) was applied to the cells and incubated for 1 h at 37 °C. After 1 h, fluorescence was measured using a Spectramax M3 multimode microplate reader (Molecular Devices, San Jose, CA, USA) with excitation and emission at 485 nm and 530 nm. Cell viability was expressed as a percentage compared to cells treated only with HBSS.

### 3.8. Caco-2-HT29-MTX-E12 Co-Culture on Transwells

Caco-2 (180,000 cells/cm^2^) and HT29-MTX-E12 (20,000 cells/cm^2^) cells, in a final volume of 200 μL, were seeded onto Costar Transwell^R^ plates to achieve a 9:1 ratio. Cells were cultured for 3 weeks in growth media at 37 °C and 5% CO_2_ to facilitate cell differentiation and produce a polarized cell barrier. Growth media was changed every 2–3 days during the 3-week differentiation period. Transepithelial electrical resistance (TEER) was measured using the Millicell-ERS Voltohmmeter from Millipore (Burlington, MA, USA) to monitor cell differentiation and formation of an intact and polarized monolayer.

### 3.9. Intestinal Absorption Assay

On the day of transport, TEER was measured to assess integrity of the cell monolayers. After ensuring TEER values were greater than 330 ohms.cm^2^, growth media was replaced with HBSS in the apical and basolateral chambers for 2 h to deplete the cells of FBS and enhance uptake of the applied digesta, as previously explained by Osborne et al. [74]. Non-cytotoxic intestinal digesta (I60; 1:2 dilution), ferrous sulfate (FeSO_4_; positive control), zinc sulfate (ZnSO_4_; positive control), and empty digesta samples were prepared in HBSS and mixed with 8 μL 8 mM ascorbic acid (final 200 μL volume) before being added to apical chambers. Next, HBSS (600 μL) was added to all basolateral chambers and treated cells were incubated at 37 °C for 2 h. After 2 h, TEER values of all chambers were measured before all apical and basolateral samples were collected and stored at −80 °C. Following this, HBSS was added to both apical and basolateral chambers and incubated overnight (16 h) for ferritin measurement (Section 3.10). After 16 h, TEER values were measured. The transport rate of minerals, trace elements, and selected phytochemicals across the Costar Transwell^R^ membrane was determined by calculating the apparent permeability coefficient (*P*app) in cm/s as explained [56].

(2) $$Papp=\frac{\Delta Q}{\Delta t}\times \frac{1}{A\;\times \;C_{0}}$$

ΔQ/Δt is steady-state appearance rate of a compound in the basolateral compartment (μmol/s).

A is the surface area of the filter (i.e., 0.33 cm^2^).

C_0_ is the initial concentration in the apical compartment (μM).

### 3.10. Ferritin Measurements

Intestinal uptake of iron was analyzed through cellular ferritin production in response to filtered (10 kDa) intestinal digesta using the Caco-2 and HT29-MTX-E12 transwell membrane model. TEER values were measured after 16 h of the intestinal absorption assay. HBSS in the apical and basolateral chambers was removed, and cells in apical chambers were washed with 200 μL phosphate-buffered saline (PBS). Trypsin (200 μL) was added to cells (apical chamber) and incubated for 5 min at 37 °C. Cells were collected from the monolayer and transferred into 1.5 mL centrifuge tube containing 400 μL growth media, followed by centrifugation at 18,800× *g* for 5 min. Cell pellets were collected after removing supernatants. 100 μL of 1× cell extraction buffer PTR (ab200018, abcam, Waltham, MA, USA) was added into the tubes containing cell pellets. The resulting cell lysates were vortexed and incubated on ice for 20 min before being centrifuged at 18,800× *g* for 20 min at 4 °C to remove debris. Human ferritin ELISA kits (ab108837, abcam, Waltham, MA, USA) were used according to the manufacturer’s protocol to determine ferritin production in diluted cell lysate supernatants.

### 3.11. Preparation and Analysis of Digesta Samples

#### 3.11.1. Sample Preparation for Elemental Composition (Minerals and Trace Elements)

For the plant samples, around 3.0–4.0 g of well-ground samples were heated in a muffle furnace (Labec, Laboratory Equipment Pty Ltd., Marrickville, NSW, Australia) at 250 °C for two hours, followed by 500 °C for 12 h to burn off the samples. The ashed samples were cooled down in a desiccator and then weighed to calculate the loss upon ignition. Around 0.1 g of ash was transferred into a clean screw-cap Teflon vessel, followed by digestion with 3 mL 15.8 M HNO_3_ and 0.5 mL 30% H_2_O_2_ at 140 °C overnight using a hot plate. Following overnight digestion, the solution was dried on a hot plate at 100 °C to near dryness. The dried samples were then re-dissolved with 1 mL 15.8 M HNO_3_, diluted with 5 mL Milli-Q water, and left to homogenize on a hot plate at 140 °C overnight. The digested samples were weighed and further diluted with Milli Q water to 1000× and 4000× appropriately for analyses of major and trace elements, respectively.

The digested gastric and intestinal digesta were centrifuged at 18,800× *g* for 15 min at 4 °C (Fresco 21 Microcentrifuge, Thermo Fisher Scientific, Schwerte, Germany). The decanted and filtered supernatants were separated and made up to 5–10 mL with 2% nitric acid prior to further elemental analysis.

#### 3.11.2. Sample Analysis

The samples were measured for major element composition using a Perkin Elmer 8300 Inductively Coupled Plasma Optical Emission Spectrometer (ICP-OES) (Perkin Elmer, Waltham, MA, USA). For analysis of trace element composition, the Agilent 7900 Inductively Coupled Plasma Mass Spectrometer (ICP-MS) (Agilent Technologies Inc., Santa Clara, CA, USA) was employed at the Environmental Geochemistry Laboratory, School of Earth and Environment Sciences, The University of Queensland (St. Lucia, QLD, Australia). The elemental composition of the samples was calibrated against the multielement calibration standards in the range of 0.1–50 ppm (for ICP-OES analysis) and 0.1–50 ppb (for ICP-MS analysis), respectively. Prior to analysis, a run sequence was created in an order that starts with the calibration standards, followed by the samples bracketed with monitor solutions and ended with the calibration standard as previously explained by Yu et al. [75] with modifications. The ICP instruments were set up and tuned to achieve optimal conditions. During the analysis, the solution was withdrawn from the sample tubes and systematically mixed with the internal standard solution containing Sc, Y, and Lu (for ICP-OES analysis) and containing ^6^Li, ^61^Ni, ^103^Rh, ^115^In, ^187^Re, and ^235^U (for ICP-MS analysis), to monitor the instrumental drift and any mass bias during the analysis. The elemental signals are detected and calibrated to obtain the elemental composition of the samples. The data quality was assessed based on the recoveries of the multielement standards measured as unknowns and those of two certified standard reference materials (CRMs): tomato leaves (SRM 1573a) and peach leaves (SRM 1547) of the NIST (National Institute of Standards and Technology, Gaithersburg, ME, USA) sourced by Sigma Aldrich (Castle Hill, NSW, Australia).

### 3.12. Statistical Analysis

The results from triplicate experiments were shown as mean ± standard deviation (SD) or mean ± standard error of the mean (SEM). Significant differences between sample means were analyzed using SPSS (version 27; IBM, Sydney, NSW, Australia) through ANOVA and Duncan’s multiple range tests. A *p*-value of 0.05 or less was considered statistically significant.

## 4. Conclusions

The present study provides crucial information about in vitro protein digestibility as well as the in vitro bioaccessibility and intestinal absorption characteristics of essential minerals and trace elements in saltbush and samphire, two important Australian indigenous halophytes. The findings from this study indicate that samphire protein is more digestible than saltbush protein, despite having a lower total protein content. Although the Fe in saltbush was found to be more bioaccessible and soluble than in samphire, the intestinal Fe absorption from samphire was higher than that in saltbush. This may have been due to the phytic acid:Fe ratio being lower in samphire compared to saltbush. Therefore, further research into the impact of non-heme Fe absorption enhancers and inhibitors is essential to maximize Fe absorption from halophytes and halophyte-based foods. In addition, in vivo studies involving healthy human subjects are also needed to substantiate the in vitro findings of the present study.

## Figures and Tables

**Table 1:** Amino acid profile of samphire and saltbush.

	Samphire	Saltbush
Essential amino acids (mg/g DW)		
Histidine *	1.2	2.4
Threonine *	2.2	4.5
Lysine *	2.6	6.3
Valine *	2.8	5.7
Isoleucine *	2.2	4.7
Leucine *	3.7	7.8
Phenylalanine *	2.3	5.2
Methionine *	ND	0.8
Non-essential amino acids (mg/g DW)		
Alanine	2.8	5.3
Proline	2.3	5.6
Tyrosine	1.4	3.0
Serine	2.6	4.6
Arginine	2.5	5.1
Glycine	3.4	5.7
Aspartic acid	4.8	9.5
Glutamic acid	5.6	11.1
Total Amino acids	42.5	87.3

* Essential amino acids; ND: not detected; Data are means (*n* = 2).

**Table 2:** Percentage changes in primary amines.

	Samphire	Saltbush	Samphire-Food	Saltbush-Food
G30	−0.2	0.1	0.3	0.0
G60	6.9	5.8	14.4	14.8
I30	133.3	72.2	73.5	117.6
I60	174.7	104.5	214.7	141.6
I120	196.6	137.7	118.4	129.8

Percentage increase in primary amines released across all sampling time points was calculated compared to G30.

**Table 3:** Apparent permeability (*P*app) of minerals and trace elements from halophyte and halophyte test food digesta.

Samples	Na *P*app (× 10^−6^ cm/s)	K *P*app (× 10^−6^ cm/s)	Mg *P*app (× 10^−6^ cm/s)	Zn *P*app (× 10^−6^ cm/s)	Fe *P*app (× 10^−6^ cm/s)
Samphire	10.4 ± 1.7 ^a^	11.4 ± 1.4 ^b^	2.1 ± 0.2 ^a^	2.4 ± 0.3 ^b^	1.0 ± 0.3 ^a^
Samphire-Food	10.8 ± 2.4 ^a^	26.0 ± 1.6 ^c^	4.5 ± 0.4 ^b^	0.9 ± 0.2 ^a^	1.0 ± 0.2 ^a^
Saltbush	5.0 ± 1.1 ^a^	4.4 ± 0.0 ^a^	1.5 ± 0.1 ^a^	1.5 ± 0.1 ^a^	2.8 ± 0.3 ^b^
Saltbush-Food	3.7 ± 0.5 ^a^	35.4 ± 0.6 ^d^	3.9 ± 0.8 ^ab^	1.0 ± 0.1 ^a^	2.3 ± 0.2 ^b^

Data are means ± SEM (*n* = 6). Means with different letters within each row indicate significant (*p* < 0.05) differences.

## Data Availability

Not applicable.

## Sample Availability

Samples of the compounds are not available from the authors.

## References

[1] Glenn E.P., Brown J.J., Blumwald E. (1999). Salt tolerance and crop potential of halophytes. Crit. Rev. Plant. Sci..

[2] Panta S., Flowers T., Lane P., Doyle R., Haros G., Shabala S. (2014). Halophyte agriculture: Success stories. Environ. Exp. Bot..

[3] Flowers T.J., Colmer T.D. (2008). Salinity tolerance in halophytes. New Phytol..

[4] Parida A.K., Das A.B. (2005). Salt tolerance and salinity effects on plants: A review. Ecotoxicol. Environ. Saf..

[5] Srivarathan S., Phan A.D.T., Hong H.T., Chua E.T., Wright O., Sultanbawa Y., Netzel M.E. (2021). *Tecticornia* sp. (Samphire)—A Promising Underutilized Australian Indigenous Edible Halophyte. Front. Nutr..

[6] Srivarathan S., Phan A.D.T., Hong H.T., Netzel G., Wright O.R.L., Sultanbawa Y., Netzel M.E. (2023). Nutritional composition and anti-nutrients of underutilized Australian indigenous edible halophytes—Saltbush, Seablite and Seapurslane. J. Food Compost. Anal..

[7] Stein A. (2010). Global impacts of human mineral malnutrition. Plant Soil.

[8] Fleet J.C., Replogle R., Salt D.E. (2011). Systems Genetics of Mineral Metabolism. J. Nutr..

[9] Thompson B., Amoroso L. (2014). Improving Diets and Nutrition: Food-Based Approaches.

[10] Pešić M.B., Milinčić D.D., Kostić A.Ž., Stanisavljević N.S., Vukotić G.N., Kojić M.O., Gašić U.M., Barać M.B., Stanojević S.P., Popović D.A. (2019). In vitro digestion of meat- and cereal-based food matrix enriched with grape extracts: How are polyphenol composition, bioaccessibility and antioxidant activity affected?. Food Chem..

[11] Akter S., Addepalli R., Netzel M., Tinggi U., Fletcher M., Sultanbawa Y., Osborne S. (2021). In vitro Bioaccessibility and Intestinal Absorption of Selected Bioactive Compounds in Terminalia ferdinandiana. Front. Nutr..

[12] Filbido G.S., Narita I.M.P., de Oliveira Pinheiro A.P., da Cruz e Silva D., Ferreira B.A., Nascimento E., Villa R.D., de Oliveira A.P. (2021). In vitro bioaccessibility of minerals in fortified infant foods and correlation between mineral absorption facilitators and inhibitors. J. Food Meas. Charact..

[13] Bertin R.L., Maltez H.F., Gois J.S.d., Borges D.L.G., Borges G.d.S.C., Gonzaga L.V., Fett R. (2016). Mineral composition and bioaccessibility in Sarcocornia ambigua using ICP-MS. J. Food Compost. Anal..

[14] Khouzam R.B., Pohl P., Lobinski R. (2011). Bioaccessibility of essential elements from white cheese, bread, fruit and vegetables. Talanta.

[15] Do Nascimento da Silva E., de Farias L.O., Cadore S. (2018). The total concentration and bioaccessible fraction of nutrients in purées, instant cereals and infant formulas by ICP OES: A study of Dietary Recommended Intakes and the importance of using a standardized in vitro digestion method. J. Food Compost. Anal..

[16] Minekus M., Alminger M., Alvito P., Ballance S., Bohn T., Bourlieu C., Carrière F., Boutrou R., Corredig M., Dupont D. (2014). A standardised static in vitro digestion method suitable for food—An international consensus. Food Funct..

[17] Hur S.J., Lim B.O., Decker E.A., McClements D.J. (2011). In vitro human digestion models for food applications. Food Chem..

[18] De Oliveira Gonçalves T., Filbido G.S., de Oliveira Pinheiro A.P., Pinto Piereti P.D., Dalla Villa R., de Oliveira A.P. (2020). In vitro bioaccessibility of the Cu, Fe, Mn and Zn in the baru almond and bocaiúva pulp and, macronutrients characterization. J. Food Compost. Anal..

[19] Kosińska-Cagnazzo A., Diering S., Prim D., Andlauer W. (2015). Identification of bioaccessible and uptaken phenolic compounds from strawberry fruits in in vitro digestion/Caco-2 absorption model. Food Chem..

[20] Cairns M.T., Gupta A., Naughton J.A., Kane M., Clyne M., Joshi L. (2017). Glycosylation-related gene expression in HT29-MTX-E12 cells upon infection by Helicobacter pylori. World J. Gastroenterol..

[21] Rozema J., Schat H. (2013). Salt tolerance of halophytes, research questions reviewed in the perspective of saline agriculture. Environ. Exp. Bot..

[22] Srivarathan S., Phan A.D.T., Wright O., Sultanbawa Y., Netzel M.E., Cozzolino D. (2021). The Measurement of Antioxidant Capacity and Colour Attributes in Wild Harvest Samphire (*Tecticornia* sp.) Samples Using Mid-infrared Spectroscopy. Food Anal. Methods.

[23] Srivarathan S., Phan A.D.T., Wright O., Cozzolino D., Sultanbawa Y., Netzel M.E. (2022). Saltbush (*Atriplex* sp.). Handbook of Phytonutrients in Indigenous Fruits and Vegetables.

[24] Abbeddou S., Rihawi S., Hess H.D., Iñiguez L., Mayer A.C., Kreuzer M. (2011). Nutritional composition of lentil straw, vetch hay, olive leaves, and saltbush leaves and their digestibility as measured in fat-tailed sheep. Small Rumin. Res..

[25] Wright K.H., Pike O.A., Fairbanks D.J., Huber C.S. (2002). Composition of Atriplex hortensis, Sweet and Bitter *Chenopodium quinoa* Seeds. J. Food Sci..

[26] De Bhowmick G., Hayes M. (2022). In Vitro Protein Digestibility of Selected Seaweeds. Foods.

[27] Hall A.E., Moraru C.I. (2022). Comparative effects of high pressure processing and heat treatment on in vitro digestibility of pea protein and starch. NPJ Sci. Food.

[28] Manditsera F.A., Luning P.A., Fogliano V., Lakemond C.M.M. (2019). Effect of domestic cooking methods on protein digestibility and mineral bioaccessibility of wild harvested adult edible insects. Food Res. Int..

[29] Habiba R.A. (2002). Changes in anti-nutrients, protein solubility, digestibility, and HCl-extractability of ash and phosphorus in vegetable peas as affected by cooking methods. Food Chem..

[30] Nestares T., López-Frías M., Barrionuevo M., Urbano G. (1996). Nutritional assessment of raw and processed chickpea (*Cicer arietinum* L.) protein in growing rats. J. Agric. Food Chem..

[31] Alain Mune Mune M., Nyobe E.C., Bakwo Bassogog C., Minka S.R. (2016). A comparison on the nutritional quality of proteins from *Moringa oleifera* leaves and seeds. Cogent Food Agric..

[32] Darragh A.J., Moughan P.J. (2019). The Effect of Hydrolysis Time on Amino Acid Analysis. J. AOAC Int..

[33] Skylas D.J., Blanchard C.L., Quail K.J. (2017). Variation in nutritional composition of Australian mungbean varieties. J. Agric. Sci..

[34] Hawas U.W., El-Kassem L.T.A., Shaher F.M., Al-Farawati R., Ghandourah M. (2022). Phytochemical Compositions of Some Red Sea Halophyte Plants with Antioxidant and Anticancer Potentials. Molecules.

[35] Sánchez-Faure A., Calvo M.M., Pérez-Jiménez J., Martín-Diana A.B., Rico D., Montero M.P., Gómez-Guillén M.d.C., López-Caballero M.E., Martínez-Alvarez O. (2020). Exploring the potential of common iceplant, seaside arrowgrass and sea fennel as edible halophytic plants. Food Res. Int..

[36] Nasir F.A., Batarseh M., Abdel-Ghani A.H., Jiries A. (2010). Free amino acids content in some halophytes under salinity stress in arid environment, Jordan. CLEAN Soil Air Water.

[37] Min J.-G., Lee D.-S., Kim T.-J., Park J.-H., Cho T.-Y., Park D.-I. (2002). Chemical Composition of *Salicornia herbacea* L. Prev. Nutr. Food Sci..

[38] Repo-Carrasco R., Espinoza C., Jacobsen S.-E. (2003). Nutritional value and use of the Andean crops quinoa (*Chenopodium quinoa*) and kañiwa (*Chenopodium pallidicaule*). Food Rev. Int..

[39] Monirujjaman M., Ferdouse A. (2014). Metabolic and physiological roles of branched-chain amino acids. Adv. Mol. Biol..

[40] Marolt G., Gričar E., Pihlar B., Kolar M. (2020). Complex Formation of Phytic Acid With Selected Monovalent and Divalent Metals. Front. Chem..

[41] Khoja K.K., Aslam M.F., Sharp P.A., Latunde-Dada G.O. (2021). In vitro bioaccessibility and bioavailability of iron from fenugreek, baobab and moringa. Food Chem..

[42] Aspuru K., Villa C., Bermejo F., Herrero P., López S.G. (2011). Optimal management of iron deficiency anemia due to poor dietary intake. Int. J. Gen. Med..

[43] D’Imperio M., Montesano F.F., Serio F., Santovito E., Parente A. (2022). Mineral Composition and Bioaccessibility in Rocket and Purslane after Zn Biofortification Process. Foods.

[44] Gera T., Sachdev H.S., Boy E. (2012). Effect of iron-fortified foods on hematologic and biological outcomes: Systematic review of randomized controlled trials. Am. J. Clin. Nutr..

[45] Joshi V., Thatte P., Prakash J., Jyothi Lakshmi A. (2014). Effect of oilseed protein concentrates and exogenous amino acids on the dialysability of iron and zinc. LWT Food Sci. Technol..

[46] Bodiga S., Krishnapillai M.N. (2007). Concurrent repletion of iron and zinc reduces intestinal oxidative damage in iron- and zinc-deficient rats. World J. Gastroenterol..

[47] Hemalatha S., Platel K., Srinivasan K. (2007). Zinc and iron contents and their bioaccessibility in cereals and pulses consumed in India. Food Chem..

[48] Cámara F., Amaro M.A., Barberá R., Clemente G. (2005). Bioaccessibility of minerals in school meals: Comparison between dialysis and solubility methods. Food Chem..

[49] Carbonaro M., Grant G., Mattera M., Aguzzi A., Pusztai A. (2001). Investigation of the mechanisms affecting Cu and Fe bioavailability from legumes. Biol. Trace Elem. Res..

[50] Anderson J.J.B. (2000). Minerals. Krause’s Food Nutrition and Diet Therapy.

[51] Maares M., Haase H. (2020). A Guide to Human Zinc Absorption: General Overview and Recent Advances of In Vitro Intestinal Models. Nutrients.

[52] WHO (2012). Report of the Formal Meeting of Member States to Conclude the Work on the Comprehensive Global Monitoring Framework, Including Indicators, and a Set of Voluntary Global Targets for the Prevention and Control of Noncommunicable Diseases.

[53] NHRMC (2017). Nutrient Reference Values for Australia and New Zealand Including Recommended Dietary Intakes.

[54] Zimmermann M.B., Chaouki N., Hurrell R.F. (2005). Iron deficiency due to consumption of a habitual diet low in bioavailable iron: A longitudinal cohort study in Moroccan children. Am. J. Clin. Nutr..

[55] Hurrell R., Egli I. (2010). Iron bioavailability and dietary reference values. Am. J. Clin. Nutr..

[56] Hubatsch I., Ragnarsson E.G.E., Artursson P. (2007). Determination of drug permeability and prediction of drug absorption in Caco-2 monolayers. Nat. Protoc..

[57] Artursson P., Karlsson J. (1991). Correlation between oral drug absorption in humans and apparent drug permeability coefficients in human intestinal epithelial (Caco-2) cells. Biochem. Biophys. Res. Commun..

[58] Lombardi-Boccia G., Carbonaro M., Lullo G.D., Carnovale E. (1994). Influence of protein components (G1, G2 and albumin) on Fe and Zn dialysability from bean (*Phaseolus vulgaris* L.). Int. J. Food Sci. Nutr..

[59] Yun S., Habicht J.-P., Miller D.D., Glahn R.P. (2004). An In Vitro Digestion/Caco-2 Cell Culture System Accurately Predicts the Effects of Ascorbic Acid and Polyphenolic Compounds on Iron Bioavailability in Humans. J. Nutr..

[60] Miller D.D., Berner L.A. (1989). Is solubility in vitro a reliable predictor of iron bioavailability?. Biol. Trace Elem. Res..

[61] Chadare F.J., Linnemann A.R., Hounhouigan J.D., Nout M.J., Van Boekel M.A. (2009). Baobab food products: A review on their composition and nutritional value. Crit. Rev. Food Sci. Nutr..

[62] Piskin E., Cianciosi D., Gulec S., Tomas M., Capanoglu E. (2022). Iron Absorption: Factors, Limitations, and Improvement Methods. ACS Omega.

[63] Abdullah M., Jamil R.T., Attia F.N. (2022). Vitamin C (ascorbic acid). StatPearls.

[64] Hurrell R.F. (2004). Phytic acid degradation as a means of improving iron absorption. Int. J. Vitam. Nutr. Res..

[65] Teucher B., Olivares M., Cori H. (2004). Enhancers of iron absorption: Ascorbic acid and other organic acids. Int. J. Vitam. Nutr. Res..

[66] Glahn R.P., Lee O.A., Yeung A., Goldman M.I., Miller D.D. (1998). Caco-2 Cell Ferritin Formation Predicts Nonradiolabeled Food Iron Availability in an In Vitro Digestion/Caco-2 Cell Culture Model. J. Nutr..

[67] Beard J.L., Dawson H., Piñero D.J. (1996). Iron metabolism: A comprehensive review. Nutr. Rev..

[68] Alvarez-Hernandez X., Nichols G.M., Glass J. (1991). Caco-2 cell line: A system for studying intestinal iron transport across epithelial cell monolayers. Biochim. Biophys. Acta BBA Biomembr..

[69] Gangloff M.B., Lai C., Van Campen D.R., Miller D.D., Norvell W.A., Glahn R.P. (1996). Ferrous iron uptake but not transfer is down-regulated in Caco-2 cells grown in high iron serum-free medium. J. Nutr..

[70] Smyth H. (2010). Defining the Unique Flavours of Australian Native Foods.

[71] Brodkorb A., Egger L., Alminger M., Alvito P., Assunção R., Ballance S., Bohn T., Bourlieu-Lacanal C., Boutrou R., Carrière F. (2019). INFOGEST static in vitro simulation of gastrointestinal food digestion. Nat. Protoc..

[72] Ménard O., Bourlieu C., De Oliveira S.C., Dellarosa N., Laghi L., Carrière F., Capozzi F., Dupont D., Deglaire A. (2018). A first step towards a consensus static in vitro model for simulating full-term infant digestion. Food Chem..

[73] Nielsen P., Petersen D., Dambmann C. (2001). Improved method for determining food protein degree of hydrolysis. J. Food Sci..

[74] Osborne S., Chen W., Addepalli R., Colgrave M., Singh T., Tran C., Day L. (2014). In vitro transport and satiety of a beta-lactoglobulin dipeptide and beta-casomorphin-7 and its metabolites. Food Funct..

[75] Yu K.-F., Kamber B.S., Lawrence M.G., Greig A., Zhao J.-X. (2007). High-precision analysis on annual variations of heavy metals, lead isotopes and rare earth elements in mangrove tree rings by inductively coupled plasma mass spectrometry. Nucl. Instrum. Methods Phys. Res. Sect. B Beam Interact. Mater. At..

